# Abiotic conditions can modify the penetrance of transgene-based lethality systems for insect population control

**DOI:** 10.1098/rspb.2025.0307

**Published:** 2025-07-02

**Authors:** Fernan Rodrigo Pérez Gálvez, Alfred M. Handler, Daniel Hahn, Justin P. Bredlau, Nicholas M. Teets

**Affiliations:** ^1^ Department of Entomology, University of Kentucky, Lexington, KY, USA; ^2^ USDA-ARS Center for Medical Agricultural and Veterinary Entomology, Gainesville, FL, USA; ^3^ Entomology and nematology, University of Florida, Gainesville, FL, USA

**Keywords:** conditional lethality, phenotypic plasticity, sterile insect technique, Tet-off gene switch, GMO risk assessment

## Abstract

Modern genetic biocontrol techniques for insect pest management, when compared to chemical insecticide spraying, offer high species specificity and reduced environmental impact, and some of these methods require the environmental release of genetically modified (GM) insects. Because organisms exposed to different environments often show variability in phenotype and gene expression, it is likely that GM insects will also experience environmentally mediated variation, potentially compromising pest control efficiency. This study examines the impact of temperature and nutrition on the early embryonic Tet-off conditional lethality system in *Drosophila melanogaster*. By independently manipulating parental and offspring environments, we assessed how exposure to variable environments influenced the probability of larval hatching and the transcript abundance of the transgenic system. Our findings revealed that: (i) transgene performance distinctly responds to temperature and nutrition; (ii) thermal stress has a greater impact when embryos, rather than parents, are exposed; and (iii) extreme nutritional conditions can markedly reduce the penetrance of transgenic lethality. Although changes in transgene transcript abundance were observed across environments, these changes did not fully explain the phenotypic variation, suggesting that factors downstream of transcription probably drive variation in transgenic lethality.

## 1. Background

Many biological traits are a function of both genotype and environment and often complex interactions between the two [1]. The same may be true for genetically engineered traits because the host organism and its transgenic system can exhibit environmental dependency. Indeed, environmental conditions can influence transgenic traits in genetically modified (GM) plants [2,3] and animals [4,5] used in agricultural contexts. Potential environment/transgene interactions are of particular interest for applications where the genetic modification is designed to be lethal to the host organism, like those used for autocidal pest control [6]. These strategies are designed to be self-limiting and prevent persistence of GM organisms in the environment. However, if environmental factors undermine transgenic lethality and increase unintended survival, it may compromise the effectiveness of the pest control programme and present regulatory challenges.

Over the past two decades, several transgenic approaches in insects, including lethal transgenes, have been developed to improve pest management programmes related to the sterile insect technique (SIT) [7]. The purpose of these transgenic approaches is to enhance the effectiveness of SIT with genetically engineered traits, for instance by introducing the expression of a fluorescent protein as a visible marker to improve the ability to distinguish between local and factory-released insects during monitoring efforts [8,9] or by introducing transgenes that improve the mating performance of males [10,11]. However, transgenic systems inducing conditional lethality are the most promising application, given their potential to replace conventional, radiation-based insect sterilization [12].

An ideal transgenic lethality system that can replace radiation-based sterilization in SIT programmes must have a dominant effect, and when expressed, induce lethality in every case (i.e. complete penetrance). The tetracycline-off (Tet-off) repressor system is the most common regulatory mechanism for conditional lethality systems in insects due to its strong induction of the gene of interest, its simple regulation of gene expression (as a genetic switch) and low levels of ‘leaky’ baseline expression that minimize potential fitness reductions during mass rearing [13]. Two configurations of Tet-Off conditional lethality have been studied for SIT application: the binary driver/effector system [14] and the single-element system named RIDL (Release of Insects Carrying a Dominant Lethal) [15]. While both systems confer dominant lethality, differences in their design probably influence their relative performance. The lethality induced by RIDL is thought to be due to exhaustion of the transcriptional machinery and/or protein degradation pathways [16], and failure rates under laboratory conditions are around 2–4% [15]. On the other hand, binary Tet-Off can have variable modes of action and typically rely on induction of specific cellular signals to cause lethality (e.g. [5,17,18]), and early systems report much lower failure rates, often well below 1% [5,19]; although see our discussion below on the importance of the definition of ‘failure’. In addition to potential differences in performance, binary Tet-Off systems offer the potential to generate early embryonic lethality that can be beneficial for agricultural pests that cause feeding damage [17].

Regardless of design, potential external factors that influence the efficacy of Tet-Off lethality systems require evaluation to inform predictions on how these systems will behave in natural environments. The probability of failure can be conceptualized from a framework of quantitative trait variation, which in natural systems is usually considered to be the product of genetic, environmental and developmental factors. Genetic factors, including the genomic position of transgene insertion [20,21] and the genetic background of the host [22], are known to introduce significant variation in the penetrance of transgenic lethality. Furthermore, because the expression and activity of lethal transgenic systems depend on the host’s cellular machinery, perturbation of these cellular processes by environmental conditions may also increase the likelihood of failure events and increase survival of transgenic insects in the field. Under these dynamic cytological environments, fluctuations in the penetrance of transgenic phenotypes are likely to be due to changes in transgene expression or other downstream processes, such as post-translational modifications to effector proteins. In some cases, environmentally induced phenotypic variation is due to adaptive plasticity that has evolved to maintain homeostasis under challenging conditions, and this plasticity may also influence phenotypes in subsequent generations [23]. However, our understanding of how environmental conditions may affect transgene trait penetrance is currently limited to a few examples [2–5], and general rules for understanding environmental effects on transgene penetrance have yet to be described.

A clearer understanding of transgene/environment interactions can provide relevant information for improving transgenic insect strains, as well as the development of a methodological framework to assess the potential risks associated with incomplete penetrance of transgenic traits in the field. Here, we used the common vinegar fly *Drosophila* (*Sophophora*) *melanogaster* to study the influence of temperature and nutritional quality on the penetrance of conditional lethality induced by a Tet-off system in the driver/effector binary configuration. Specifically, we tested a construct designed for early embryonic expression driven by the *serendipity alpha* (*sryα*) promoter sequence 1 [24] with a resultant lethal effect specified in the effector element by *head involution defective* Alanine-5 (*hid*^Ala5^) [25], the phosphorylation-desensitized mutant of the endogenous pro-apoptotic *hid* gene [26]. This construct is designed for agricultural pests that cause feeding damage, where early embryonic lethality is desired, and is distinct from the RIDL system used in mosquitoes.

We exposed embryos carrying these constructs or their parents to a range of temperatures and diets to identify the extent to which environmental conditions affect the penetrance of transgenic lethality. We expected offspring survival under these conditions to be related to the transcript abundance of transgene components, because the penetrance of developmental mutations is often the result of variable transcription (e.g. [27,28]). Our results show that while transgenic lethality remained high under most treatments evaluated, (i) environmental conditions at the extremes of permissible abiotic conditions can decrease the penetrance of transgenic lethality, (ii) the norm of reaction for transgenic lethality depends on both the type (i.e. temperature versus nutrition) and timing (i.e. exposure in parents versus offspring) of environmental variation, and (iii) transcript abundance of the transgene is insufficient to explain reduced penetrance of the transgenic lethality trait, suggesting that environmental variation in penetrance is acting through mechanisms downstream of transcript abundances such as translation or post-translational modification.

## 2. Methods

### (a) Transgenic system and animal husbandry

We studied the transgenic *D. melanogaster* strain Double Homozygous-1 (DH-1) that carries a binary driver/effector Tet-off transgenic system that induces lethality during early embryogenesis by ectopic expression of a mutant pro-apoptotic factor [29]. DH−1 is in the mutant *white*^1118^ genetic background and was created via inbreeding two strains carrying independently inserted *piggyBac* vectors containing either the driver or the effector cassettes. The driver vector, pB{PUb-DsRed.T3, s1-tTA}, contains *tTA* regulated by the *sryα* s1 promoter sequence and the DsRed.T3 fluorescent protein regulated by the constitutive *polyubiquitin* (*PUb*) promoter. The effector vector, pB{3xP3-ECFP-5’HS4>5’HS4-TREp-hidAla5-5’HS4>5’HS4}, encodes the pro-apoptotic protein HID^Ala5^ regulated by the TRE sequence, a heptameric tandem repeat of the tetracycline operator sequence [13]. Both *hid*^Ala5^ and TRE are flanked by 5’HS4 insulator sequences, and the construct also includes Enhanced Cyan Fluorescent Protein (ECFP) regulated by the 3xP3 promoter. The Tet-off system is induced in the syncytial blastoderm during cellularization between 120 and 170 min at 22°C after fertilization [30]. In the absence of tetracycline, tTA dimers bind to TRE in the effector element, induce the ectopic expression of *hid*^Ala5^ and, consequently, activate *hid*-dependent programmed cell death resulting in embryonic lethality. When present, tetracycline molecules allosterically inhibit dimerization of tTA monomers and prevent their induction of *hid*^Ala5^ expression. We applied 64 ug ml^−1^ tetracycline to the rearing media of DH-1 to maintain the strain and ensure flies were exposed to sufficient levels of antibiotics to suppress the system.

In addition, we used the wild-type *D. melanogaster* Oregon-R strain as the simulated target pest population. Specifically, we crossed adult unmated Oregon-R females with transgenic DH-1 males in a tetracycline-free environment to simulate the mating events intended in the autocidal strategy in the field. To determine the effect of our environmental treatments on the probability of hatching in wild-type flies, we also conducted parallel experiments with Oregon-R flies exposed to the same environmental conditions.

### (b) Embryo collection

We simulated the most common SIT strategy where only males are released, and therefore we evaluated embryos carrying a single paternally inherited copy of the transgenic system. Because no alleles of the *piggyBac* insertion loci are present in the wild-type genome, the transgenic system exists in these embryos in a hemizygous state. For the remainder of the text, we will refer to embryos hemizygous for the lethal transgene as ‘transgenic’ and those with unmodified genotype as ‘wild-type’. We quantified the efficacy of transgenic lethality as the probability of hatching in transgenic embryos, all of which were expected to die because the parental generation was not supplied with tetracycline after adult emergence (as would occur in the field). In addition, we evaluated the probability of wild-type embryos hatching under the same experimental treatments as a control to determine the general effects of our treatments. The calculated probability of hatching is derived from binary data (hatch/no hatch) and can be interpreted as the hatching rate often used in agricultural science and SIT literature [31].

Transgenic and wild-type embryos were reared under the following standard protocol. Baseline conditions were 25°C, 12 : 12 L : D cycle and a cornmeal-yeast-molasses diet. These conditions were modified accordingly for each experimental treatment as described below. Parental flies were sorted by sex using brief CO_2_ anaesthesia 1−2 h after adult eclosion and kept on tetracycline-free food vials for five days. On the fifth day after adult emergence, 200 adult males and 200 adult females were allowed to mate for 24 h at 25°C in embryo collection cages (Genesee Scientific Corp., El Cajon, CA, USA) containing grape agar (Genesee Scientific Corp., El Cajon, CA, USA) and a small amount of baker’s yeast paste to promote oogenesis and oviposition. On the 6th day after adult emergence, freshly oviposited embryos were collected from the oviposition cages and incubated for scoring.

To estimate the probability of hatching, embryos were harvested in 1 h intervals using a collection basket fitted with nylon mesh (Genesee Scientific Corp., El Cajon, CA, USA), then washed with 75% ethanol for 5 seconds, and rinsed with distilled water. These embryos were incubated in Petri dishes on black filter paper (Ahlstrom Munksjo, Mt Holly Springs, PA, USA) and saturated with distilled water until hatching or death. To estimate transcript abundance, embryo samples were collected in 30 min periods and incubated for 150 min to allow embryos to reach the late blastoderm stage, except in experiments where embryonic temperature was varied, in which the timing was adjusted (see below). After completing the incubation period, embryos used for measuring gene expression were recovered from the oviposition substrate with a fine paint brush, suspended in 30 µl of distilled water inside 1.5 ml screwcap tubes, and frozen to −80°C. In baseline conditions, sampling time was 165 min on average, which was chosen as the sampling point between the induction of the Tet-off lethality system and the end of blastoderm cellularization to provide a transcriptional snapshot of both driver and effector elements in the same biological sample.

### (c) Experimental treatments

**Figure 1.**
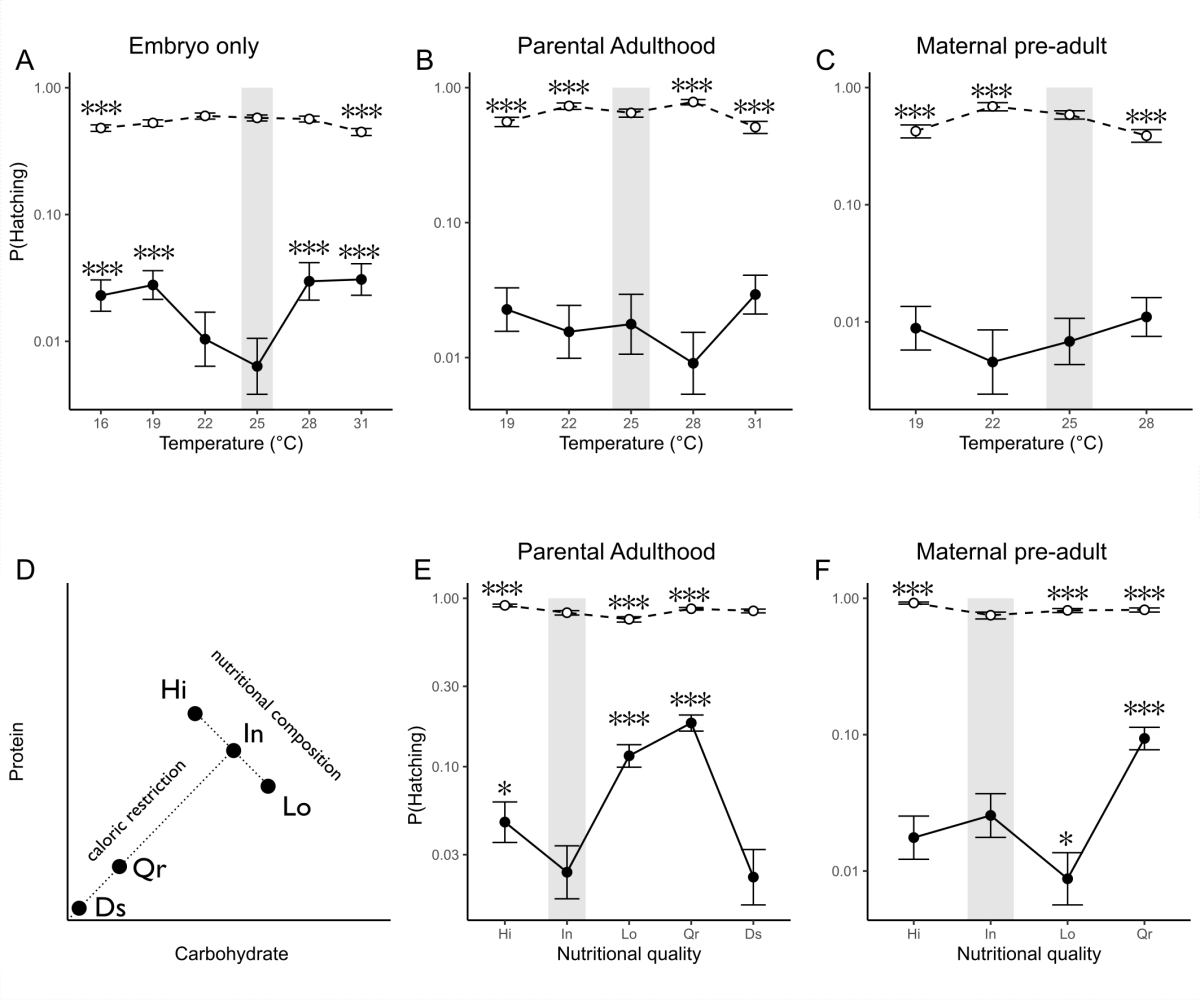
Hatching reaction norms to (A–C) temperature and (E–F) nutritional quality treatments for transgenic (solid lines, closed circles) and wildtype embryos (dashed lines, open circles). Treatments: (A) direct embryo exposure, (B,E) parental exposure during adulthood, (C,F) maternal development exposure. (D) Nutritional quality treatments are shown in the geometric space of nutrition with an isocaloric line or a caloric restriction dotted line. Pairwise differences in post hoc tests are Z-test ratios (*p‐value < 0.05; **p‐value < 0.01; ***p‐value < 0.001) within genotype, with reference to treatments in shaded boxes (25°C for temperature, Equal P : C for nutrition). Error bars represent the upper and lower confidence limits estimated from the contrast’s odds ratios.

Our experimental design quantified incomplete penetrance of transgenic lethality in distinct thermal and nutritional environments experienced at three different life stages of exposure. For both thermal and nutritional experiments, the influence of abiotic treatments was determined relative to a pre-selected baseline condition: 25°C for temperature and Intermediate protein : carbohydrate (P : C) diet (see description below) for nutrition experiments. In the case of our thermal manipulations, multiple temperatures were selected at regular intervals across the permissive temperature range for growth of *D. melanogaster* (12–32°C) [32]. Using the geometric framework of nutrition [33], we designed a series of nutritional-quality treatments across the P : C space in two axes: first, isocaloric diets with varying P : C ratios (High, Intermediate, Low P : C), and second, dietary restriction treatments with reduced caloric content (Intermediate P : C, Quarter, Desiccation) (figure 1D). The specific treatments applied to each life stage are described in detail below.

**Figure 2.**
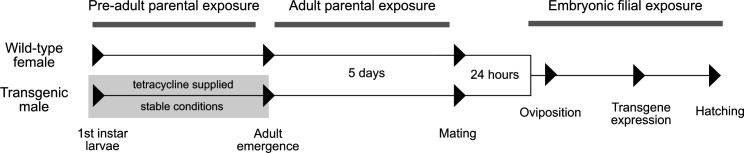
Experimental design of chronic exposure stages. Pre-adult parental exposure to treatments was experienced exclusively by the mother from larva stage to adult emergence. This is followed by adult parental exposure, where sex-sorted adult flies of were exposed to treatments for 5 days. Afterward, males and females are held together in baseline conditions for 24 h to mate. Finally, embryonic filial exposure spans from embryo oviposition to hatching or death. Filled triangles represent the milestones used to determine stages.

Both transgenic and wild-type flies experienced the above-mentioned environmental conditions in one of three predetermined life stages (figure 2). We defined the filial embryonic stage as the period experienced by the embryos from oviposition until death or larval hatching. Nutritional quality treatments were omitted for our embryo-only treatments given that embryos do not feed and exclusively depend on their parental nutritional supply. Next, the parental adulthood stage was defined as the period when both wild and released transgenic adult parents are in the field together. We also varied environmental conditions during the maternal pre-adult stage, from the first larval instar to adult eclosion, to simulate environments wild-type females may experience during immature stages. For these experiments, transgenic males were maintained at standard laboratory conditions during development to simulate rearing facilities.

Each experimental treatment consisted of one abiotic condition applied to a specific exposure stage. For the embryo-only treatments, embryos were exposed to one of six temperatures (16, 19, 22, 25, 28 and 31°C) from the time of oviposition to hatching or death. Because the rate of embryogenesis changes with temperature, we adjusted the time of phenotyping and transcript abundance sampling using a temperature-dependent developmental rate equation for *D. melanogaster* [34]:


$$t_{Dmel}=4.02e^{37.31/T},$$

where *t* is development time in hours and *T* is temperature in degrees Celsius. The incubation period for gene expression samples was calculated as the time equivalent to 180 min (30 min collection + 150 min treatment) at 25°C to determine the temperature-specific stage of late blastoderm cellularization. Similarly, for the estimation of the probability of hatching, we calculated a time interval equivalent to 24 h at 25°C to ensure all treatments had sufficient time to complete embryogenesis.

Experimental conditions for adult-only treatments were applied for 5 days between adult emergence and mating of the parental flies, but mating was always performed at 25°C to avoid potential confounding effects with changes in mating behaviour [35]. For adult-only exposure, five temperatures were evaluated (19, 22, 25, 28 and 31°C). Five levels were also evaluated for nutritional quality, namely three levels of isocaloric diets that differ in the P : C ratio (High P : C 7.4:7.2, Intermediate P : C 4.7:11.4, and Low P : C 2.0:15.7) as used by Matzkin *et al*. [36], one caloric restriction level named ‘Quarter’ (P : C 1.6:5.4) equivalent to 25% of caloric content of the Intermediate P : C diet, and one ‘Desiccation’ level that consisted of preventing access to water and food for 24 h prior to mating.

We also investigated the effect of temperature shock in parental adults on conditional lethality. Thermal shock treatments consisted of 1 h exposure to either 0°C (cold-shock) or 34°C (heat-shock), and these were compared to a no-shock treatment kept at 25°C. Thermal shock treatments were applied to 6 days old parental adults of both genotypes just after the 24 h mating period. Embryo samples for the estimation of the probability of hatching were collected at 1, 2, 4, 24 and 48 h after thermal shock. Transcript abundance samples were collected at 2 and 24 h after thermal shock.

For the maternal juvenile development conditions, four temperatures (19, 22, 25 and 28°C) and four nutritional quality levels (High P : C, Intermediate P : C, Low P : C, Quarter) were applied from the first instar of the maternal larval stage to maternal adult emergence, after which females were kept at 25°C. Transgenic parental males were developed in baseline conditions (25°C and cornmeal–yeast–molasses diet).

### (d) Phenotyping transgenic lethality

A hatching event was scored when an embryo presented a visibly disrupted chorion. This hatching definition identifies individuals that survive early embryonic lethality, serving as a stringent measure of the lethal transgenic system’s performance. Developed mouth hooks and coordinated movements are required for first instar *D. melanogaster* larva to break the vitelline membrane and chorion [37], hence disruption of these layers indicates that the expected transgene mode of action (i.e. early embryonic lethality) was disrupted. Furthermore, our measurement of lethality is amenable to high-throughput screening, because dishes of eggs can be photographed and scored afterwards, as opposed to direct observations of live larvae.

### (e) Estimation of transgene expression

To estimate expression levels of the transgenic system across our experimental conditions, we measured the transcript abundance of *tTA*, *hid*^Ala5^ and the housekeeping gene *RP49* as an endogenous control with real-time qPCR using the primer sequences provided in electronic supplementary material, table S1. Frozen embryos were homogenized in lysis buffer with a Fast-Prep24 TM 5G Bead Homogenizer (MP Biomedical, Santa Ana, CA, USA) with 40−60 0.5 mm zirconia/silica beads (Biospec Products, Bartlesville, OK, USA). RNA was extracted with the RNeasy mini kit (Qiagen, Germantown, MD, US) following the manufacturer’s protocol, and cDNA was synthesized from 100 ng RNA using the QScript cDNA Synthesis Kit (QuantaBio, Beverly, MA, USA). Real-time qPCR reactions were prepared with ten-fold dilutions of the cDNA using PerfeCTa SYBR Green Fastmix (QuantaBio, Beverly, MA) and were measured in triplicate on a QuantaStudio 6 Flex real-time PCR System (Applied Biosystems, Foster City, CA, USA) following instructions provided by the manufacturer. Melting curves were inspected for single peaks to ensure no primer dimers were generated. The primers designed for each gene demonstrated good linearity (*R*^2^ > 0.99) and efficiency (1.98 < *E* < 2.05) using an 8-point standard curve. Fold changes of *tTA* and *hid*^Ala5^ were calculated with the $2^{-\Delta \Delta CT}$ method [38] using *RP49* as the reference gene.

### (f) Replication design and sample sizes

A total of six experiments were conducted: three involving chronic exposure to temperature treatments at distinct life stages, two involving the chronic exposure to nutritional treatments at distinct life stages, and one involving thermal shock in adults. Each of the first five experiments that included chronic exposure was repeated across two independent cohorts, while the thermal shock experiment was performed three times. Each experiment was performed as a weekly sequence of random blocks consisting of three different temperature or nutrition treatments. Treatments were applied to cohorts of two oviposition cages, one per embryo genotype. From each oviposition cage, samples for probability of hatching were taken at three sequential one-hour embryo collections that varied in size (electronic supplementary material, table S2). Given that each treatment was performed twice with three embryo samples collected on each occasion, the replication design in the five experiments with chronic exposure included a total of six biological replicates per treatment per genotype. In the thermal shock experiment, a single sample was collected per cohort producing a total of three biological samples per treatment per sampling time (except for 48 h treatments which only had two observations).

For gene expression in the first five chronic exposure experiments, two samples of 20 embryos were taken at 30 min intervals for two experimental replicates, totalling four biological replicates per treatment. Occasionally one outlier was removed leaving three observations. In the thermal shock experiment, one sample of 20 embryos per treatment (no-shock, heat-shock and cold-shock) was recovered at 2 h and 24 h evaluation times at 30 min intervals for each of the three experimental replicates, resulting in three biological replicates per group.

### (g) Statistical analyses

All statistical analyses were performed in R v. 4.1.0 [39] with lme4 (v. 1.1−29) and emmeans (v. 1.8.0) and graphed with ggplot2 (v. 3.5.1). The data for hatching rate and transcript abundance are publicly available and hosted on Dryad [40]. The probability of hatching was estimated with logistic regression using a generalized mixed effects model in each of the six experiments by fitting the joint dataset of transgenic and control embryos. The fixed effects in the five chronic exposure experiments were *Genotype* and *Temperature* (or *Nutrition* depending on the experiment) and their interaction. The random effects were modelled with a repeated measures approach using random intercepts for the three sequentially collected samples (termed *Repeated Measure*) and random slopes for *Cohort*. The fixed effects in the thermal shock experiment model were *Genotype*, *Shock* (hot, cold, no shock), *Time after shock* and their interaction. The random effects were also modelled with a repeated measures approach using random intercepts for *Time after shock* and random slopes for *Replicate*. In each model, the explanatory variable of interest, either *Temperature*, *Nutrition quality* or *Shock*, was modelled as a nominal categorical variable.

To determine which treatments were different from standard laboratory conditions, after fitting linear models (electronic supplementary material, table S3), we used custom contrasts to define post hoc pairwise comparisons between experimental and reference conditions. Specifically, we applied Dunnet’s test approach to assess differences between treatments and controls in a generalized mixed model. The ‘control’ treatments used were 25°C and ‘Intermediate P : C’ diet for thermal and nutritional experiments, respectively. In the case of thermal shock, the ‘control’ treatment used for pairwise comparisons was *No-shock* at each time point evaluated. In each case, p-values were adjusted for multiple comparisons using the multivariate t method.

To analyse differences in transcript abundance in the five chronic exposure datasets, we fitted a categorical linear model using ΔCT values as a function of treatment levels (*Temperature* or *Nutrition*). Pairwise differences of transcript abundance were calculated relative to a reference treatment, 25°C for temperature and Intermediate P : C diet for nutritional quality treatments. Significant differences between the treatment and the control groups were determined with Dunnet’s post hoc test [41]. For the parental shock treatments, transcript abundance was analysed using a linear model with the variables *Time after shock*, *Shock* and their interaction. Significance was assessed in *Heat shock* versus *No-shock* and *Cold shock* versus *No-shock* pairwise comparisons for each *Time after shock* level with a post-hoc test using custom contrasts.

## 3. Results

In this study, we quantified the efficacy of an early embryonic conditional lethality system in *D. melanogaster* across a range of temperature and nutritional treatments. When analysed independently, the probability of transgenic embryo hatching across all baseline replicates (25°C, Intermediate P : C, No-shock) varied from 0.4% to 2.9%, and was 1.76% on average (s.d. = 1.79, *n* = 44) with a median of 1.29%, indicating some variation in the efficacy of the lethal transgene across replicates under standard laboratory conditions. Nonetheless, in many cases, our environmental treatments had a much larger effect on lethality than the variation present in the controls (see below).

Certain temperature treatments applied directly to the embryos reduced the penetrance of transgenic lethality compared to the reference treatment at 25°C (electronic supplementary material, table S4). In this case, the probability of failure displayed an inverted performance curve with a minimum probability of hatching of 0.64% at 25°C [s.e. = 0.1]) but as high as 2.78% [s.e. = 0.36] and 3.00% [s.e. = 0.4] at 19°C and 31°C treatments, respectively (figure 1A). In contrast, temperature treatments applied to parental adults or across maternal development had no effect on lethality in transgenic insects (figure 1B,C). For wild-type embryos, many of the temperature treatments changed the probability of hatching relative to 25°C, especially for treatments applied to parental adults or during maternal development (figure 1A–C). In most cases, these treatments reduced survival relative to 25°C.

Modifying the nutritional environment of the parental flies also affected the penetrance of transgenic lethality (electronic supplementary material, table S5). Individuals fed the Quarter-strength diet (1.8 P : 4.5 C versus Intermediate P : C ‘control’ 7.1 P : 17.9 C) either as adults or as maternal larvae had decreased penetrance of transgenic lethality (figure 1E,F). Specifically, adult parental exposure to the Quarter-strength diet increased the probability of failure from 2.31% [s.e. = 0.42] in the reference diet to 16.43% [s.e. = 0.8], while pre-adult maternal exposure increased failure of embryonic lethality from 2.88% [s.e. = 0.48] to 8.16% [s.e.= 0.48].

Beyond the effects of the caloric-restriction diet, isocaloric imbalanced P : C diets influenced the penetrance of transgenic lethality (figure 1E,F). Relative to the reference Intermediate P : C diet (2.31%, s.e. = 0.42) of parental adulthood exposure, both High P : C (3.92%, s.e. = 0.53) and Low P : C (11.16%, s.e. = 0.82) diets significantly decreased the penetrance of transgenic lethality following parental adulthood exposure (electronic supplementary material, table S5). On the other hand, Low P : C significantly increased the penetrance of transgenic lethality and reduced probability of hatching to 0.70% (s.e. = 0.15) when experienced exclusively during maternal pre-adult development, relative to the reference diet, which had a probability of hatching of 2.88% (s.e. = 0.48).

**Figure 3.**
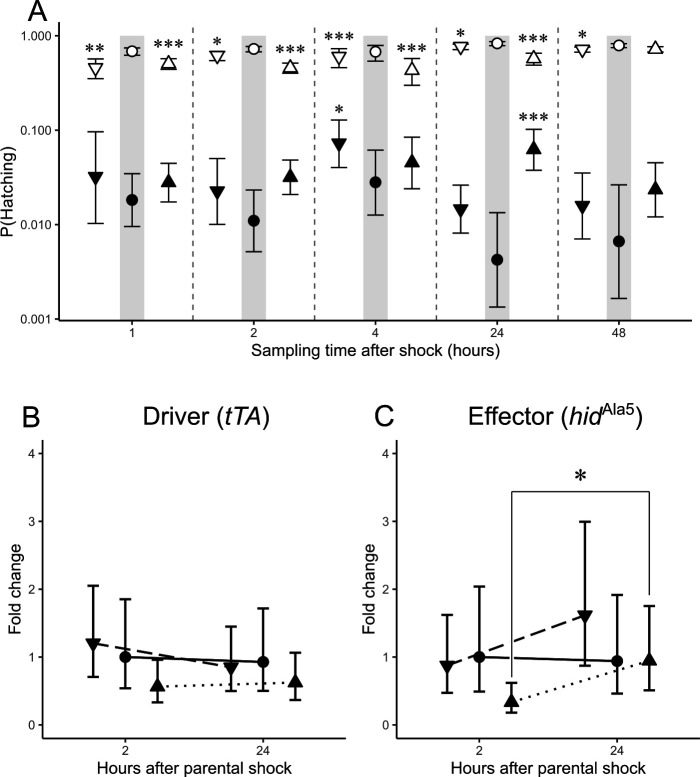
Effect of parental adult thermal shock on the penetrance of transgenic lethality. Probability of hatching (A) was determined for each treatment (No-shock: circles, cold-shock: downward triangles, heat-shock: upward triangles) per genotype (wild-type: empty shapes, transgenic: solid shapes). Transcript abundance of the driver (B) and effector (C) elements of the transgenic system were evaluated at 2 and 24 h after shock. Pairwise differences are assessed between sampling times within each shock treatment with custom contrasts (see §g for details). Error bars represent the upper and lower confidence limits estimated from the contrast’s odds ratios (A) or standard errors (B,C). Long dashed lines indicate differences between cold-shock, dotted lines between heat-shock and solid lines between no-shock estimates (*p‐value < 0.05; **p‐value < 0.01; ***p‐value < 0.001).

We further studied the influence of parental exposure to heat (34°C) and cold (4°C) shock treatments to identify potential cross-generational impacts on transgenic lethality. Our results indicate that parental thermal shock influenced the survival of both transgenic and wild-type embryos (figure 3A). Both parental shock treatments significantly reduced the penetrance of transgenic lethality with a modest effect size at just a single time point after exposure (electronic supplementary material, table S6). The effect of cold shock was apparent in transgenic embryos 4 h post-shock, with an 8.5% (s.e. = 1.4) probability of hatching compared to 3.1% (s.e. = 1.0) in the no-shock control. In turn, heat shock induced a 6.2% (s.e. = 1.5) probability of hatching in transgenic embryos collected 24 h post-shock, compared to 0.4% (s.e. = 0.2) in the no-shock treatment. The transient influence of thermal shock in the probability of hatching of transgenic embryos was distinct from that in wild-type embryos, where the first embryo collection after shock presented the largest decrease in the probability of hatching and slowly recovered over time (figure 3A).

**Figure 4.**
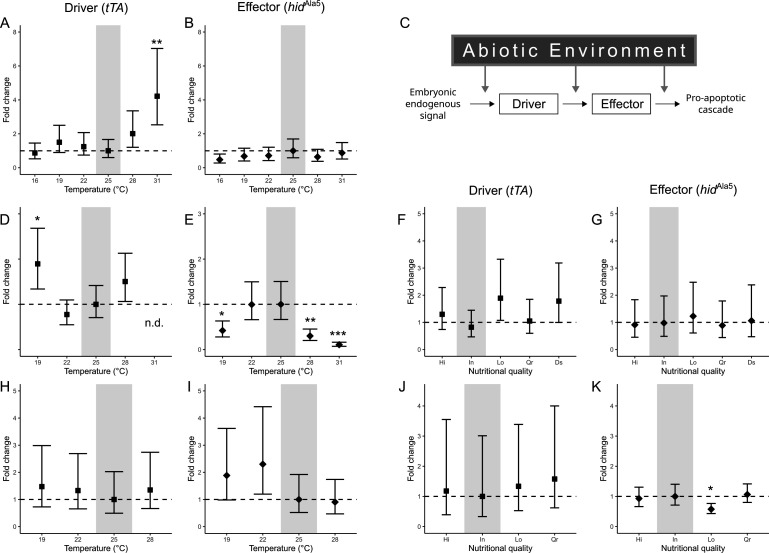
Gene expression of the transgenic conditional lethality system in response to temperature and nutritional quality. Treatments were applied directly to embryos (A,B), to parents during adulthood (D,E,F,G), and to the mother during larval development (H,I,J,K). The transgenic lethality system (C) is induced by an early embryonic endogenous signal, followed by the expression of its elements to induce a pro-apoptotic cascade. Significant differences were estimated with *t*‐test ratios. Error bars represent the upper and lower confidence limits estimated from the contrast’s standard errors. Baseline conditions used as reference for pairwise differences (25°C for temperature, Equal P : C for nutrition) are indicated with a grey box, and the ideal expectation of no change in gene expression is indicated with a horizontal dashed line (*p‐value < 0.05; **p‐value < 0.01; ***p‐value < 0.001). Tetracycline transactivator gene: *tTA*; head involution defective Alanine5 gene: *hid*^Ala5^.

We hypothesized that variation in the penetrance of transgenic lethality would be at least partly due to interference in the molecular events required for the complete expression and function of the lethal transgene (figure 4C). We tested this hypothesis at the transcriptional level by measuring the transcript abundance of *hid*^Ala5^ (driver) and *tTA* (effector) in the transgenic embryos. In general, there was little relationship between transcript abundance and observed lethality, with the following exception. Embryos subjected to pre-adult maternal exposure to Low P : C ratio diet (figure 4K) showed a decrease in penetrance of transgenic lethality together with a decrease in transcriptional abundance of the lethal transgene *hid*^Ala5^ when compared to Intermediate P : C diet (t(9.25)=2.9, *p* = 0.04).

While most of the transcriptional abundance results did not show significant changes across treatments, distinct patterns in transgene expression were observed in thermal treatments experienced directly by the embryos or by their parents during adulthood (figure 4). As mentioned above, this variation in transcript abundance did not track the observed variation in lethality. For example, with embryonic exposure to 31°C, the penetrance of transgenic lethality decreased (figure 1A and electronic supplementary material, table S4), but the transcript abundance of *tTA* increased (figure 4A), each relative to the 25°C control treatment. Furthermore, there was also significant variation in transgene expression in embryos following parental adulthood exposure (figure 4D,E), even though no significant differences were observed in the penetrance of transgenic lethality (figure 1B). For example, a decrease in transcript abundance for the effector element *hid*^Ala5^ was observed at 19°C and 31°C, while *tTA* either increased (19°C) or became undetectable (31°C).

In accordance with the transient influence of thermal shock in the penetrance of transgenic lethality, the transcript abundance of *hid*^Ala5^ significantly increased in response to heat shock (figure 3C), which induced a 2.8-fold increase between 2 and 24 h embryo collections (ΔΔCT = 1.5, t (6)=2.8, *p* = 0.029). As in previous results, the direction of the varying transgene expression was opposite to our predictions because the transcript abundance of *hid*^Ala5^ increased over time while the penetrance of transgenic lethality decreased during that interval (figure 3A).

## 4. Discussion

In this study, we evaluated the effects of the abiotic environment on the penetrance of an early embryonic Tet-off conditional lethality system intended for autocidal genetic biocontrol. As expected, the performance of the transgenic system was high under optimal conditions, but our results indicate that some suboptimal thermal or nutritional scenarios can modify the penetrance of the transgenic lethal phenotype. Specifically, suboptimal temperature treatments experienced for the duration of embryonic development, or suboptimal nutrition treatments experienced by the adult parental generation or during maternal development, increased the probability of survival relative to baseline in 8 out of 19 conditions tested, while in our thermal shock treatments, the probability of survival increased in only 2 out of 10 groups sampled. Interestingly, a decrease in the probability of hatching (and by extension an increase in efficiency of transgenic lethality) was observed in a single occasion, after maternal pre-adult exposure to low P : C diet. Taken together, our findings suggest that experiencing chronic environmental stress can increase a transgene’s likelihood of failure.

For conditions that influenced penetrance, the strongest reductions in embryonic lethality were typically observed in the most extreme treatments. In addition, the influence of the abiotic environment intensified with more recent exposure, with the strongest effects of temperature occurring in the embryonic stage and the strongest effects of nutrition in the parental adults. In the case of embryonic temperature exposure, the penetrance of transgenic lethality decreased at both high and low temperatures, essentially an inverse thermal performance curve. In contrast, the probability of hatching in wild-type embryos displayed the canonical shape of a thermal performance curve, with the highest probability of hatching near 25°C and then decreasing at the higher and lower ends of the temperature range [42]. It is also worth noting that in our baseline controls, which were collected repeatedly over several months during the study, there was some variation in lethality across replicates, suggesting that even subtle changes in the lab environment can influence lethality. Nonetheless, even when considering this variation within our control group, we clearly detected environmentally mediated reductions in penetrance across many of our treatments.

Previous studies have also suggested that temperature can affect the penetrance of transgenic systems. Horn & Wimmer [5] evaluated the same conditional lethality system as ours at three temperatures and counted the number of active larvae. Like our study, the penetrance of transgenic lethality was temperature-dependent, but exhibited a negative correlation between survival and temperature (highest survival at 18°C, no survival at 29°C). Our criteria of penetrance (early embryonic lethality) is distinct from the moving larvae used by Horn & Wimmer [5], which may partly explain the difference in results. Our study also used a slightly different genetic construct, which included 95 additional base pairs in the *sryα* promoter sequence (electronic supplementary material, s1 instead of s2) [24] and lacked flanking 5′HS4 insulator elements in the driver cassette present in Horn & Wimmer [5]. These differences could lead to variations in the temperature-dependence of the penetrance of lethality.

Besides confirming a transgene/environment interaction in response to temperature, our results suggest that parental nutrition, particularly caloric restriction, can cause cross-generational variation in the penetrance of transgenic lethality. Understanding the connection between nutrition and transgenic lethality is highly relevant because resource limitation is prevalent in natural environments [43]. Most notably, the largest decreases in the penetrance of transgenic lethality resulted from caloric restriction treatments during the adult parental and maternal pre-adult stages. Dietary restriction induces physiological and biochemical changes in insects, including increases in lifespan [44]. Caloric restriction in adults also enhances tolerance to heat shock, oxidative stress, and starvation in progeny [45], suggesting that these stress responses could influence transgenic phenotypes in embryos. In our experiments, isocaloric diets above and below optimal P : C ratio also influenced the penetrance of transgenic lethality, and as with caloric restriction, previous work has demonstrated that macronutrient composition can influence key life history traits including lifespan and reproduction (see [46]). However, the contribution of each macronutrient to transgenic penetrance may be confounded by our experimental protocol, because when a diet is available *ad libitum*, ingestion of unbalanced foods is often compensated by increased or decreased feeding to meet the required intake of a critical nutrient (usually protein), leading to over or under ingestion of other nutrients and decreased fitness [47]. Thus, our results indicate that future research on specific facets of diet composition may prove fruitful in identifying mechanisms behind transgene variation due to nutrition.

One of the primary motivations for our study was to develop performance standards for transgenic insect strains that can be used to improve these technologies and facilitate data-driven environmental risk assessment. However, limitations of lab-based methods must be considered when extrapolating to natural environments. When applied to risk forecasting purposes, different definitions of transgene failure may lead to divergent conclusions. For instance, if larval lethality remains high at elevated temperatures, as indicated by the lack of physiological escapers reported in Horn & Wimmer [5], only environments with lower temperatures would have an increased risk of failure. On the contrary, the inverted u-shaped performance curve for temperature observed in our experiments suggests a higher risk of failure in both cooler and warmer environments or seasons. Therefore, our study highlights the need for precise definitions of performance when comparing transgenic systems, even those with the same mode of action.

We hypothesized that environmental conditions would affect transgene penetrance by modulating transcriptional activation of the transgene components, but our results provide minimal support for this hypothesis. For the most part, there was no relationship between phenotypic variation in penetrance of embryonic lethality and transcript abundance of the driver and effector components (i.e. one element increased while the other decreased or showed no change). Due to the large number of treatments in our study, we only sampled embryos at a single time point for gene expression, and perhaps environmental conditions disrupted the timing of expression. Thus, the expected relationship between transgene expression and penetrance may be more apparent if embryos were sampled on a finer temporal scale. Nonetheless, there are multiple functional aspects of the lethality system that we did not assay (induction time, cellular localization, potential interaction with endogenous elements, etc.) that may explain the reduced penetrance of transgenic embryonic lethality, but these thorough mechanistic experiments were beyond the scope of the current work.

While thorough molecular characterization of the induction of a conditional lethality system across a range of environments could provide insights for improvement, it may not fully explain the environmentally induced plasticity of transgenic lethality. This is because the penetrance of the pro-apoptotic effector gene depends on both the expression of the recombinant protein as well as its interaction with endogenous elements participating in the apoptotic cascade. Given that in our study transcript levels of the transgene components were not well correlated with penetrance of lethality, modulation of the expression and/or activity of other apoptosis machinery is a plausible mechanism for environmentally induced plasticity in embryonic death. Several genetic elements directly interact with *hid,* including *Drosophila* inhibitor of apoptosis 1 (*DIAP1*) gene, which is negatively regulated by HID in the control of caspase-dependent apoptosis [48]. Another candidate is Caspase-8, which is necessary for HID-induced programmed cell death, and it is activated by direct interaction with HID, such that deficiency of Caspase-8 (*dredd*) in flies suppresses HID-induced apoptosis [49,50]. Indeed, several components of apoptosis signalling are modulated by temperature and other stressors (e.g. [51,52]), providing a likely mechanism by which environmental variation influences the activity of the *hid*^Ala5^ transgene. We acknowledge any discussion of molecular mechanism is speculative at this point, but the lack of relationship between transcript abundance and transgene penetrance should stimulate additional experiments to identify molecular mechanisms that contribute to transgene performance in natural environments.

## 5. Conclusions

Predicting the probability of failure of a transgenic lethal system in open field conditions is crucial for the environmental risk assessment of GM organisms. Ideally, performance estimates generated in standard laboratory conditions would predict the failure risk for a planned release programme, but our results show that environmental conditions that insects often encounter can alter the efficiency of transgenic lethality systems. This discovery highlights the complexity of the genetically engineered autocidal strategy, which involves interactions between host genome, transgenes, biochemical and cellular mechanisms, and the environment [53]. It remains to be seen whether the observed environmentally induced plasticity of the evaluated transgenic system is a widespread phenomenon across the catalogue of transgenic insect strains intended for environmental release. In particular, the conditional lethality systems that operate with the positive feedback loop of RIDL technology, some of which have been commercialized for mosquito control and tend to have higher failure rates than early embryonic lethality (e.g. [15,54,55]), may have distinct responses to changing environmental conditions. Thus, there is a clear need to further investigate the environmental component of transgene function, as our results conclusively indicate that the abiotic environment can induce physiological changes that alter the potency of lethal transgenes.

## Data Availability

The data for hatching rate and transcript abundance are publicly available and hosted on Dryad [40]. Supplementary material is available online [56].
